# Association of Cognitive Function and Water Polo Exposure with Sports Injuries in Youth Water Polo Players

**DOI:** 10.3390/jfmk11040411

**Published:** 2026-10-07

**Authors:** Tea Galić, Ana-Marija Vukas, Frane Mihanović, Neven Kovačević

**Affiliations:** 1 Department of Prosthodontics, Study of Dental Medicine, School of Medicine, University of Split, Šoltanska 2A, 21000 Split, Croatia; 2 University Hospital of Split, 21000 Split, Croatia; 3 Croatian Water Polo Federation, 10000 Zagreb, Croatia; nevenkovacevic57@gmail.com; 4 Study of Dental Medicine, School of Medicine, University of Split, 21000 Split, Croatia; anamarijatucak@gmail.com; 5 Faculty of Health Sciences, University of Split, 21000 Split, Croatia; frane.mihanovic@ozs.unist.hr; 6 Faculty of Kinesiology, University of Split, 21000 Split, Croatia

**Keywords:** water polo, cognitive functions, sports injuries, head injuries, orofacial injuries

## Abstract

**Background:** Water polo is a sport that combines substantial physical contact with high cognitive demands, exposing athletes to sports-related injuries, including head and orofacial trauma. This study investigated associations between cognitive performance, cumulative water polo exposure and injury history in youth water polo players. **Methods:** This cross-sectional study included 161 youth water polo players (103 males, 58 females; mean age 15.76 ± 1.53 years). Sports-related injury history was assessed using an online questionnaire. Cognitive performance was evaluated with the computerized Stroop test. Sport exposure time (years of official water polo participation) and number of games played were extracted from the official Croatian Water Polo Federation database. Logistic regression analyses were adjusted for age and sex; additional head injury models were adjusted for years of participation. **Results:** A history of overall sports-related, head and orofacial injury was reported by 93 (57.8%), 65 (40.4%) and 51 (31.7%) players, respectively. Participants with head injury history had lower Stroop OffTime (55.90 ± 6.46 s vs. 58.56 ± 8.72 s, *p* = 0.028) and OffTime + OnTime (118.63 ± 16.10 s vs. 125.10 ± 22.11 s, *p* = 0.033) in unadjusted comparisons. However, the Stroop test measure was not independently associated with head injury history after adjustment. Each additional year of water polo participation was associated with 34% higher odds of reporting head injury history (aOR = 1.34, 95%CI 1.08–1.65, *p* = 0.007), while number of games played was not independently associated with any injury history outcomes. **Conclusions:** Within the limits of the cross-sectional design, longer participation in water polo, used as an indirect measure for cumulative exposure, appears more strongly associated with head injury history than the cognitive measures assessed. Prospective longitudinal studies are needed to establish temporal relationships and evaluate future injury risk.

## 1. Introduction

Water polo is a physically and cognitively demanding open-skill team sport characterized by intermittent high-intensity swimming, repeated changes in direction, throwing and frequent physical contact between opponents [1,2]. During gameplay, athletes are simultaneously required to monitor the ball, teammates, opponents, spatial positioning and rapidly changing tactical situations while performing complex motor actions under substantial physical demands [1,2,3,4]. Many contacts occur below the water surface and outside the referee’s field of vision and players are exposed to grabbing, holding, wrestling, collisions and ball impacts throughout training and competition [2,5,6,7]. Consequently, water polo is associated with a broad spectrum of both acute traumatic and overuse injuries [8]. Although shoulder disorders represent a substantial proportion of water polo injuries, acute injuries frequently involve the head and face, with orofacial and dental trauma representing an important component of the overall injury burden [8,9,10]. Head impacts have received particular attention because water polo combines intentional and unintentional player contacts, as well as high-velocity ball impacts. Blumenfeld et al. [11] reported that 36% of surveyed water polo players had experienced at least one concussion during their sporting career, with differences according to sex, playing position and competition level. Some previous studies have demonstrated that repetitive head impacts occur during water polo competition and that their frequency and characteristics vary according to tactical role and playing position [12]. The orofacial region is similarly exposed to direct contact with the ball, or elbows and hands of the opposing players. Zamora-Olave et al. [10] reported that 57.9% of water polo players sustained at least one orofacial injury during a competitive season, while mouthguard use remained uncommon. Despite the recognized injury burden of the sport, evidence regarding injuries and their associated factors in youth water polo players remains limited compared with that available for collegiate and adult athletes.

In addition to the physical demands of water polo, successful performance requires highly developed perceptual and cognitive abilities. Players must rapidly identify relevant stimuli, process information, select an appropriate response, inhibit competing or inappropriate actions and continuously modify their behavior according to changes in the playing environment [7,13,14,15]. Attention, information-processing speed, reaction time, working memory, cognitive flexibility and inhibitory control may therefore contribute not only to sport-specific performance but potentially also to an athlete’s ability to respond safely to unexpected situations [16]. Previous research in youth water polo has demonstrated differences in cognitive performance between athletes and non-athletic peers and suggested a relationship between cognitive abilities and sport-specific performance [14,15,17].

Increasing attention has therefore been directed toward neurocognitive performance as a potential factor associated with sports injury occurrence. In dynamic sporting situations, delayed perceptual processing, impaired attentional control, or an inability to inhibit an inappropriate motor response could interfere with the rapid generation of an appropriate movement strategy. Swanik et al. [18] found that collegiate athletes who subsequently sustained a non-contact anterior cruciate ligament injury demonstrated slower reaction times and processing speed and poorer visual and verbal memory than matched uninjured athletes. Visual-motor reaction time has also been proposed as a potentially modifiable factor associated with musculoskeletal injury risk [19]. These prospective observations provide a rationale for examining cognition in open-skill sports such as water polo, where players are required to respond to rapidly changing and often unpredictable external stimuli under substantial temporal and physical demands.

Executive functions may have particular relevance in this context. The Stroop task is commonly used to assess inhibitory control and cognitive interference while also providing information related to processing speed and attentional control. In water polo, inhibitory control may enable a player to suppress an initially prepared response when the tactical situation changes and rapidly select a more appropriate action [6,14]. However, evidence directly linking Stroop performance or other executive-function measures with sports injury history remains scarce, particularly among youth athletes and in aquatic team sports. The relationship between cognitive function and injury is also potentially bidirectional: poorer cognitive performance could theoretically precede injury, whereas head injury may temporarily or persistently affect reaction time, processing speed, memory, attention and executive functions [20]. Accordingly, the present cross-sectional study was designed to evaluate associations with injury history, not temporal sequence, causation or prediction of future injury.

Despite increasing interest in both neurocognitive factors associated with sports injury and the epidemiology of water polo injuries, little is known about the relationship between cognitive performance, cumulative sport exposure and injury history, specifically in youth water polo players. Furthermore, studies simultaneously evaluating these factors in relation to overall sports-related, head and orofacial injuries are lacking. Examining cognitive performance together with measures of exposure may therefore help clarify whether cognitive differences are independently associated with injury history or are primarily related to greater accumulated participation in the sport. The primary aim of the present study was to investigate associations between selected cognitive functions, assessed using a computerized Stroop test and a history of sports-related injuries in male and female youth water polo players, with particular emphasis on head and orofacial injuries. A secondary aim was to examine associations between cumulative water polo exposure, reflected by years of participation and number of games played, and injury history. It was hypothesized that players with a history of overall sports-related, head or orofacial injuries would demonstrate poorer cognitive performance, reflected by slower psychomotor and motor speed and weaker inhibitory control, than their uninjured peers. Greater cumulative water polo exposure was hypothesized to be associated with a higher likelihood of reporting previous injury.

## 2. Material and Methods

### 2.1. Study Design and Participants

The study was approved by the Ethics Committee of the School of Medicine University of Split (N: 2181-198-03-04-25-0028) and conducted according to the ethical standards set by the Declaration of Helsinki. All participants or their parents/legal guardians for minor participants signed a written informed consent before participation.

This cross-sectional observational study included a retrospective assessment of sports-related injury history and a concurrent evaluation of cognitive performance in youth water polo players. Inclusion required participation in Croatian Water Polo Federation training camps or national-team training programs between the 2022/2023 and 2025/2026 seasons. To maximize sample representativeness, all available eligible players were invited; no additional exclusion criteria were applied, and no a priori sample-size calculation was performed. At a two-sided significance level of alpha = 0.05, a reference-effect sensitivity analysis based on the odds ratio of 1.64 reported by Giesche et al. [21] suggested that the final sample had approximately 84% power for overall sports-related injury, 86% for head injury and 81% for orofacial injury to detect associations of comparable magnitude. This retrospective sensitivity estimate does not exclude limited power or imprecision for smaller associations or subgroup analyses. The available sample did not support reliable sex-stratified or cluster-adjusted multivariable models; analyses therefore treated participants as individual observations and included age and sex as covariates. No exercise intervention or standardized training program was implemented; consequently, exercise increments and progression rules were not applicable, and individual training load was not prospectively monitored. Participants who did not complete the study protocol were excluded (n = 11: two did not finish the Stroop test, eight did not complete the injury questionnaire and one did not provide consent). Finally, 161 participants, youth male and female water polo players, all members of youth Croatian Water Polo National Teams (U15, U16, U18, U19) were included in this study.

### 2.2. Measurements and Outcomes

#### 2.2.1. Sports Injury Assessment

Injury data on sports-related injuries was self-reported via on-line questionnaire used in previous studies [22]. Questionnaire comprised 34 questions divided in five sections—personal data, overall sports-related injuries, head injuries and orofacial injuries, including injury mechanism (contact or non-contact), number of injuries, affected region, symptoms, game/practice interruption, preventive measures. Before completing the questionnaire, the participants received detailed, clearly explained instructions from the principal investigator (TG) and had the opportunity to ask questions if anything was unclear to them. To ensure controlled conditions, possible ambiguities among the participants and to ensure peace and quiet in the room, the principal investigator (TG) was present in the room during the research. For this study, overall sports-related injuries comprised injuries to body regions other than the head and orofacial region, which were analyzed separately. The primary outcomes were binary indicators (yes/no) of a self-reported history of overall sports-related, head and orofacial injury. Because exposure hours and athlete-exposures were unavailable, incidence rates per 1000 exposure hours could not be calculated; the reported percentages represent the prevalence of injury history in the analyzed sample. Playing position, years of official water polo participation and number of official games played were obtained from the Croatian Water Polo Federation database. Years of participation and games played were considered indirect measures and did not quantify training hours, actual playing time, contact frequency or head-impact magnitude.

#### 2.2.2. Cognitive Functions Assessment

The cognitive functions assessments were instructed by three experienced investigators (TG, FM, NK) in groups of eight players and under standardized conditions (i.e., instructions, room, temperature, light, chair and test equipment). Testing was conducted at similar times of day across participants to minimize the effects of diurnal variation in cognitive functioning. All participants practiced each test during a test trial and had sufficient time to ask questions before the actual test started.

The cognitive assessment comprised the computerized EncephalApp(v 2.21 (48)) Stroop test administered on tablets and included Stroop Off and Stroop On conditions. Stroop Off primarily reflected psychomotor speed, whereas Stroop On additionally required response inhibition and motor responding. The total time needed to complete each of these conditions was documented. Shorter completion times indicate better cognitive performance and vice versa. Two derived measures were also calculated: OffTime + OnTime as a composite completion-time measure, and OnTime − OffTime as an index of additional interference-related processing and inhibitory control. These measures have been used in our previous research [14,17,23,24,25]. The Stroop test measures assess selected aspects of processing speed and inhibitory control and should not be interpreted as a comprehensive assessment of all cognitive domains relevant to sports injury.

#### 2.2.3. Statistical Analysis

Statistical analyses were performed using MedCalc for Windows, version 19.4 (MedCalc Software, Ostend, Belgium). Continuous variables were summarized as mean ± standard deviation or median with interquartile range, as appropriate, and categorical variables as frequencies and percentages. The Kolmogorov–Smirnov test was used to assess the normality of the data distribution. Differences in continuous variables between injured and non-injured groups were assessed using Welch’s independent samples *t*-test for approximately normally distributed variables and the Mann–Whitney U test for non-normally distributed variables. Differences in categorical variables were evaluated using the χ^2^-test or Fisher’s exact test in cases where the frequency was less than five.

Following previous research on cognitive predictors of injury risk [19,21,26], binary logistic regression was used to examine associations with each binary injury history outcome. Separate models were constructed for overall sports-related, head and orofacial injury history. Years of participation and number of games played were entered in separate models because they represented related indirect measure for cumulative exposure. Age and sex were selected a priori as potential confounders because both may be related to developmental stage, sport exposure, cognitive performance and injury history. The coefficients were interpreted as associations with the odds of reporting previous injury, not as estimates of incidence, causation or future injury risk.

To assess whether cognitive performance was independently associated with head injury history, additional multivariable logistic regression models were constructed with each Stroop test measure entered separately. These models were adjusted for age, sex and years of water polo participation. Separate Stroop test models limited multicollinearity among correlated Stroop-derived measures and reduced model overparameterization. Adjusted odds ratios (aORs), 95% confidence intervals (CIs) and exact *p* values were reported. Because the analyses were exploratory and the cognitive measures were correlated, no formal multiplicity adjustment was applied. Statistical significance was set at *p* < 0.05.

## 3. Results

A total of 161 youth water polo players were included in the study, with a mean age of 15.76 ± 1.53 years, 103 male (64.0%) and 58 female (36.0%) players. Regarding playing position, there were 29 (18.0%) goalkeepers, 31 (19.3%) center-defenders, 19 (11.8%) center-forwards, 52 (32.3%) drivers and 30 (18.6%) wings. Participants’ cognitive performance, sports exposure and prevalence of self-reported injury history are presented in Table 1.

Overall sports-related injuries occurred most frequently during training (37/93, 39.8%), followed by games (34/93, 36.6%); 22 players (23.6%) reported injuries in both settings. Head injuries occurred with equal frequency during training and games (23/65, 35.4% each), whereas 19 players (29.2%) reported head injuries in both settings. Among players with orofacial injuries, 32/51 (62.7%) reported injuries during games, 17/51 (33.3%) during training and 2/51 (3.9%) in both settings. Among the 91 participants with available mechanism data for overall sports-related injury, contact with another player was the predominant mechanism (54/91, 59.3%). Player contact was also reported for 42/65 (64.6%) head injuries. Multiple mechanisms were reported for 14/65 (21.5%) head injuries, most commonly player contact combined with ball contact and/or impact with the pool edge or goal. Almost all orofacial injuries resulted from contact with another player (50/51, 98.0%); one injury (1/51, 2.0%) was attributed to impact with the pool edge.

Comparisons according to injury history status are presented in Table 2. For overall sports-related injury and orofacial injury history, no significant differences were observed in age, years of water polo participation, number of games played or the Stroop test measures. Playing position was not significantly associated with overall or head injury history. In contrast, the prevalence of orofacial injury differed significantly according to the primary playing position (*p* = 0.020), with the highest prevalence observed among center-defenders (15/31, 48.4%) and center-forwards (9/10, 47.4%), followed by driver/wing players (22/82, 26.8%) and goalkeepers (5/29, 17.2%). In an exploratory adjusted analysis using goalkeepers as the reference category, center-defenders had higher odds of orofacial injury (aOR = 5.37, 95% CI 1.54–18.80, *p* = 0.008), as well as center-forwards (aOR = 4.09, 95% CI 1.06–15.86, *p* = 0.041), whereas the association for drivers and wings was not significant (aOR = 1.65, 95% CI 0.54–5.06, *p* = 0.378).

In logistic regression models adjusted for age and sex, years of water polo participation were associated with higher odds of reporting a history of head injury, whereas no significant associations were observed for overall sports-related or orofacial injury history (Table 3). Each additional year of participation was associated with 34% higher odds of head injury history (aOR = 1.34, 95% CI 1.08–1.65, *p* = 0.007). Number of games played was not independently associated with any injury history outcome. These cross-sectional associations do not establish that longer participation caused injury or predicts future injury.

In multivariable logistic regression analyses, none of the Stroop test measures was independently associated with head injury history after adjustment for age, sex and years of water polo participation (Table 4). 

Longer participation remained associated with higher odds of head injury history across the four separate Stroop models (aOR range = 1.33–1.36; 95% CI range 1.08–1.68), with *p* values from 0.005 to 0.007. Female sex was also independently associated with higher odds of head injury history than male sex (aOR range = 3.33–3.52; 95% CI range 1.58–7.38), with *p* values from <0.001 to 0.002. Age was not independently associated with head injury history. These coefficients describe adjusted associations with previously reported injury and should not be interpreted as predictive effects.

## 4. Discussion

To the best of our knowledge, this is the first study to examine associations between cognitive performance, water polo exposure and a history of overall sports-related, head and orofacial injuries in youth water polo players. Participants with head injury history had shorter Stroop OffTime and OffTime + OnTime values in unadjusted comparisons, whereas no consistent cognitive differences were observed for overall or orofacial injury history. After adjustment for age, sex and years of participation, however, Stroop test measures were not independently associated with head injury history. Longer participation in water polo remained associated with higher odds of head injury history. Thus, within this cross-sectional dataset, an indirect measure for cumulative exposure showed a more consistent association with head injury history than the cognitive measures assessed.

The association between longer water polo participation and head injury history is consistent with the specific physical demands and contact characteristics of the sport. Water polo players are repeatedly exposed to intentional and unintentional contact with opponents, as well as to ball impacts, with many contacts occurring below the water surface and outside the referee’s direct field of vision [3,4,7,27]. Blumenfeld et al. [11] reported that 36% of surveyed water polo players had experienced at least one concussion during their playing careers and that concussion prevalence increased with competitive level and accumulated playing exposure. Objective monitoring of collegiate water polo players has subsequently confirmed that repeated head impacts occur during both training and competition and that their frequency varies according to playing situation and position [12]. Furthermore, surveillance data from collegiate water polo identified the head and face as the second most frequently injured anatomical region and concussion as the most common individual injury diagnosis [8]. These observations provide a plausible explanation for the present association between years of participation and history of head injury: each additional year of involvement in water polo represents additional cumulative opportunity for player-to-player contact, ball impacts and other potentially injurious events.

Another important consideration is cumulative sport exposure. Athletes who have participated in water polo for longer periods have accumulated more training sessions, competitions, physical contacts, repetitive throwing actions and, therefore, more opportunities for head or facial impacts [8]. At the same time, prolonged sport-specific training and competitive experience may influence cognitive performance [14]. Age and sex may also be related to cognitive development, accumulated participation, reporting patterns and injury history during adolescence; they were therefore treated as potential confounders. Biological maturation could additionally influence neuromuscular and cognitive development and injury susceptibility, but maturation indicators were not available in this study. Apparent associations between cognitive measures and injury history may therefore reflect differences in age, sex, playing experience, maturation or cumulative exposure rather than an independent relationship between cognitive performance and injury. Although prospective injury surveillance ideally quantifies exposure using hours of participation or athlete-exposures, years of water polo participation and number of games played were used here as complementary, indirect measures for cumulative exposure.

Participants with head injury history initially appeared to perform faster on selected Stroop test measures, particularly OffTime, contrary to the initial hypothesis of poorer cognitive performance in injured athletes. This unadjusted difference did not remain significant after adjustment for age, sex and years of participation and should not be interpreted as evidence that head injury improves cognition or that better cognition increases injury risk. Greater sport experience may be related both to accumulated opportunities for injury and to sport-specific cognitive adaptation, but the present design cannot test this explanation. Water polo is an open-skill sport requiring continuous processing of rapidly changing visual and tactical information, inhibition of inappropriate responses, spatial awareness and rapid decision making under physical contact [5,6,13], and previous research has reported better response inhibition and psychomotor performance in youth water polo players than in age-matched non-athletes [17]. Conversely, sport-related concussion can cause transient deficits in processing speed, attention, memory and executive function, particularly in the acute phase [28], although evidence regarding persistent deficits is heterogeneous [20]. The absence of an adjusted association in the present study may reflect resolution of short-term cognitive effects before testing, heterogeneity in injury severity and timing, under-recognition of repetitive head impacts or the limited cognitive domains sampled by the Stroop test. The test primarily assesses processing speed, interference and response inhibition and may not detect changes in working memory, visual memory, complex reaction time or divided attention [25]. These explanations remain hypotheses requiring prospective testing with clinically verified injuries and repeated multidomain cognitive assessment.

Sports injuries are multifactorial events arising from interactions among intrinsic characteristics, exposure, physical demands, environmental factors and situational mechanisms. Cognitive abilities such as attention, processing speed, inhibitory control and decision making could theoretically affect responses to hazardous situations, but evidence linking baseline cognitive performance with subsequent injury remains inconsistent. Recent prospective data in professional football similarly found no significant independent association between selected cognitive functions and subsequent musculoskeletal injury, although exploratory trends were reported [21]. In the present study, the Stroop test measures were not independently associated with injury history after adjustment and therefore should not be regarded as predictors of injury. Cognitive performance was likewise not independently associated with orofacial injury history. Previous studies have shown that orofacial and dental injuries are common in water polo [9,10,22], and direct contact with another player, an elbow, a hand or the ball is a predominant mechanism [29,30]. Such mechanical and situational factors may be more relevant to orofacial trauma than individual differences in the limited cognitive domains measured in this study, although this interpretation cannot be tested directly using the present cross-sectional data.

Although both head and orofacial injury involve similar anatomical regions and may result from similar contact mechanisms, their relationship with cumulative sport exposure may differ. Head injuries include a broader range of impacts occurring during player contact and ball exposure, some of which may occur repeatedly throughout training and competition. Orofacial injuries, particularly dental trauma, may depend more strongly on the direction and location of an individual impact and on protective equipment. Thus, prevention strategies for head and orofacial injuries may need to address partly different mechanisms even when the injuries occur during the same sports situations. Despite the evidence that mouthguards reduce dentoalveolar trauma in contact sports, mouthguard use remains low among water polo players [10,22,29,30].

From a practical perspective, longer participation may identify youth water polo players with greater accumulated opportunity for head contact and a more frequent history of head injury, but it should not be interpreted as a causal risk factor on the basis of this cross-sectional study. The findings support early, sustained attention to injury surveillance and prevention throughout athlete development. Coaches, medical staff, athletes and parents should be educated about recognition and reporting of head injuries, appropriate medical evaluation and adherence to return-to-play procedures. Prospective monitoring is needed to determine whether rule, training or role modifications reduce avoidable head contact. Given the established burden of orofacial trauma in water polo, education about dental injury prevention and use of appropriately designed mouthguards should also remain a priority.

Several limitations of this study should be considered. First, the cross-sectional design precludes causal inference and temporal ordering: cognitive characteristics may have preceded injury, changed after injury or been unrelated to it. The findings therefore concern associations with injury history and not prediction of future injury. Second, injuries were reported retrospectively and are susceptible to recall and classification bias, particularly for minor or remote events; severity, exact timing, number and clinical diagnosis of head injuries were not standardized. Third, the Stroop test sampled selected aspects of processing speed and inhibitory control but not the full range of cognitive functions relevant to sports injury. Fourth, years of participation and number of games were indirect exposure measures that did not quantify training hours, actual playing time, competition intensity, athlete-exposures or the frequency and magnitude of contacts and head impacts. Accordingly, injury incidence per 1000 exposure hours could not be estimated. Fifth, biological maturation was not assessed. Age adjustment only partly addresses developmental heterogeneity and residual confounding by pubertal or neuromuscular maturation is possible. Sixth, the sample size did not permit adequately powered sex-stratified, team-clustered or position-specific confirmatory models. Treating participants as independent observations may have underestimated uncertainty if outcomes were correlated within national-team age groups or training cohorts. Seventh, several correlated cognitive outcomes and injury categories were examined without formal multiplicity correction; isolated nominal *p* values and exploratory position estimates should therefore be interpreted cautiously. Finally, participants were youth national-team water polo players, which limits generalizability to recreational youth, adult and other competitive populations.

Despite these limitations, this study simultaneously evaluated selected cognitive measures, demographic characteristics, two indirect measures for cumulative water polo exposure and three clinically relevant injury history categories in the same youth athlete sample. Reporting both years of participation and official games provided complementary, although incomplete, indicators of cumulative sport exposure. Separate adjusted Stroop test models and reporting of aORs with 95% CIs and exact *p* values allowed the independent associations to be evaluated without treating the analyses as predictive models. Separate consideration of head and orofacial injuries also reflects their potentially different mechanisms.

## 5. Conclusions

Although participants with head injury history completed selected Stroop test measures faster in unadjusted comparisons, no Stroop test measure remained independently associated with head injury history after adjustment for age, sex and years of participation. Longer participation in water polo was associated with higher odds of reporting head injury history and should be interpreted as an indirect marker of cumulative exposure rather than evidence of causation or future risk. Prospective longitudinal studies should include baseline multidomain cognitive testing, biological maturation indicators, detailed athlete exposure and training load data, objective head impact monitoring, clinically verified injuries and repeated follow-up assessments.

## Figures and Tables

**Table 1:** Participants’ characteristics, cognitive performance, sports exposure and prevalence of injury history.

Variable	Total Sample (N = 161)
Age (years)	15.76 ± 1.53
Female (n)	58 (36.0)
Male (n)	103 (64.0)
Water polo participation (years)	6.92 ± 1.99 (3–12)
Games played (n)	172.01 ± 86.44 (13–495)
Stroop OffTime (s)	57.49 ± 7.97
Stroop OnTime (s)	64.99 ± 12.82
Stroop OffTime + OnTime (s)	122.48 ± 20.10
Stroop OnTime − OffTime (s)	7.50 ± 7.18
Overall sports injury (n)	93 (57.8)
Head injury (n)	65 (40.4)
Orofacial injury (n)	51 (31.7)
Playing position	
Goalkeeper (n)	29 (18.0)
Center-defender (n)	31 (19.3)
Center-forward (n)	19 (11.8)
Driver (n)	52 (32.3)
Wing (n)	30 (18.6)

Values are presented as mean ± standard deviation (range) or number (%), as appropriate.

**Table 2:** Comparisons of youth water polo players according to injury history status.

Overall Sports-Related Injury History			
	Without Injury (N = 68)	With Injury (N = 93)	*p*
Age, years	15.68 ± 1.71	15.82 ± 1.39	0.579 *
Water polo participation, years	7 (5–8)	7 (5–8.2)	0.194 **
Games played, n	148.5 (94–213)	167 (121–227)	0.167 **
Stroop OffTime, s	57.04 ± 8.11	57.82 ± 7.90	0.541 *
Stroop OnTime, s	64.95 ± 13.02	65.02 ± 12.74	0.972 *
Stroop OffTime + OnTime, s	121.99 ± 20.48	122.85 ± 19.93	0.791 *
Stroop OnTime − OffTime, s	7.92 ± 7.16	7.20 ± 7.23	0.535 *
Female sex, n	15/68 (22.1)	43/93 (46.2)	0.002 ***
Head Sports-Related Injury History			
	Without Injury (N = 96)	With Injury (N = 65)	*p*
Age, years	15.46 ± 1.37	16.20 ± 1.66	0.003 *
Water polo participation, years	6 (5–8)	8 (5–9)	<0.001 **
Games played, n	144 (102.2–201.8)	198 (122–237.8)	0.014 **
Stroop OffTime, s	58.56 ± 8.72	55.90 ± 6.46	0.028 *
Stroop OnTime, s	66.53 ± 14.12	62.72 ± 10.29	0.050 *
Stroop OffTime + OnTime, s	125.10 ± 22.11	118.63 ± 16.10	0.033 *
Stroop OnTime − OffTime, s	7.97 ± 7.88	6.82 ± 6.01	0.296 *
Female sex, n	26/96 (27.1)	32/65 (49.2)	0.004 ***
Orofacial Sports-Related Injury History			
	Without Injury (N = 110)	With Injury (N = 51)	*p*
Age, years	15.74 ± 1.56	15.84 ± 1.58	0.704 *
Water polo participation, years	7 (5–8.8)	7 (5–9)	0.852 **
Games played, n	167 (112.5–213.8)	147.5 (120–232.5)	0.841 **
Stroop OffTime, s	57.58 ± 8.26	57.45 ± 7.68	0.924 *
Stroop OnTime, s	64.93 ± 13.11	65.47 ± 13.17	0.812 *
Stroop OffTime + OnTime, s	122.51 ± 20.73	122.92 ± 20.11	0.907 *
Stroop OnTime − OffTime, s	7.35 ± 7.09	8.02 ± 7.78	0.608 *
Female sex, n	34/110 (30.1)	24/51 (47.1)	0.070 ***

Values are presented as mean ± standard deviation, median (Q1–Q3), or number (%), as appropriate. * Welch *t*-test. *p* < 0.05. ** Mann–Whitney *U* test. *p* < 0.05. *** χ^2^ test. *p* < 0.05.

**Table 3:** Multivariable logistic regression analyses—exposure variables and injury history outcomes.

Outcome	Exposure Predictor	N	Events	aOR	95% CI	*p*	Adjustment
Overall sports injury	Years of water polo participation	161	93	1.15	0.94–1.40	0.172	Age, sex
Overall sports injury	Games played	161	93	1.16	0.94–1.43	0.180	Age, sex
Head injury	Years of water polo participation	161	65	1.34	1.08–1.65	0.007	Age, sex
Head injury	Games played	161	65	1.15	0.93–1.41	0.192	Age, sex
Orofacial injury	Years of water polo participation	161	51	1.02	0.84–1.25	0.816	Age, sex
Orofacial injury	Games played	161	51	0.95	0.77–1.18	0.651	Age, sex

OR = odds ratio; aOR = adjusted odds ratio; CI = confidence interval. aORs are expressed per 1-year increase in water polo participation and per 50-game increase in games played. Models were adjusted for age and sex. Statistical significance was set at *p* < 0.05.

**Table 4:** Multivariable logistic regression analyses—cognitive measures of head injury history after adjustment for age, sex, and years of water polo participation.

Stroop Test Model	Stroop Test Measure			Years of Water Polo Participation			Female Sex			Age		
Predictor Entered	aOR	95% CI	*p*	aOR	95% CI	*p*	aOR	95% CI	*p*	aOR	95% CI	*p*
OffTime	0.98	0.93–1.03	0.385	1.36	1.10–1.68	0.005	3.33	1.58–7.00	0.002	1.14	0.86–1.52	0.360
OnTime	0.99	0.96–1.02	0.456	1.35	1.09–1.66	0.006	3.44	1.65–7.21	0.001	1.15	0.87–1.53	0.325
OffTime + OnTime	0.99	0.97–1.01	0.409	1.35	1.09–1.67	0.005	3.40	1.62–7.12	0.001	1.14	0.86–1.53	0.357
OnTime − OffTime	0.99	0.94–1.05	0.729	1.33	1.08–1.65	0.007	3.52	1.68–7.38	<0.001	1.20	0.92–1.56	0.189

OR = odds ratio; aOR = adjusted odds ratio; CI = confidence interval. Models included one Stroop test measure at a time and were adjusted for age, sex, and years of water polo participation to avoid collinearity among Stroop-derived measures. *p* < 0.05.

## Data Availability

The data presented in this study are available on request from the corresponding author.
